# A food web approach reveals the vulnerability of biocontrol services by birds and bats to landscape modification at regional scale

**DOI:** 10.1038/s41598-021-02768-0

**Published:** 2021-12-08

**Authors:** José M. Herrera, Bruno Silva, Gerardo Jiménez-Navarro, Silvia Barreiro, Nereida Melguizo-Ruiz, Francisco Moreira, Sasha Vasconcelos, Rui Morgado, Javier Rodriguez-Pérez

**Affiliations:** 1 Mediterranean Institute for Agriculture, Environment and Development, University of Évora, Casa Cordovil, 2nd Floor, R. Dom Augusto Eduardo Nunes 7, 7000-651 Évora, Portugal; 2 CIBIO, Centro de Investigação em Biodiversidade e Recursos Genéticos, InBIO Laboratório Associado, Campus de Vairão, Universidade do Porto, 4485-601 Vairão, Portugal; 3 CIBIO, Centro de Investigação em Biodiversidade e Recursos Genéticos, InBIO Laboratório Associado, Instituto Superior de Agronomia, Universidade de Lisboa, Tapada da Ajuda, 1349-017 Lisbon, Portugal; 4 BIOPOLIS Program in Genomics, Biodiversity and Land Planning, CIBIO, Campus de Vairão, 4485-661 Vairão, Portugal; 5 Centro de Ecologia Aplicada “Professor Baeta Neves” (CEABN), InBIO, Instituto Superior de Agronomia and Universidade de Lisboa, Tapada da Ajuda, 1349-017 Lisbon, Portugal; 6 Institute for Multidisciplinary Research in Applied Biology (IMAB), Depto. Ciencias del Medio Natural, Centro Jerónimo de Ayanz, Universidad Pública de Navarra (UPNA), Campus Arrosadía, 31006 Pamplona, Spain

**Keywords:** Agroecology, Biodiversity, Community ecology, Ecosystem services

## Abstract

Pest control services provided by naturally occurring species (the so-called biocontrol services) are widely recognized to provide key incentives for biodiversity conservation. This is particularly relevant for vertebrate-mediated biocontrol services as many vertebrate species are of conservation concern, with most of their decline associated to landscape modification for agricultural purposes. Yet, we still lack rigorous approaches evaluating landscape-level correlates of biocontrol potential by vertebrates over broad spatial extents to better inform land-use and management decisions. We performed a spatially-explicit interaction-based assessment of potential biocontrol services in Portugal, using 1853 pairwise trophic interactions between 78 flying vertebrate species (birds and bats) and 53 insect pests associated to two widespread and economically valuable crops in the Euro-Mediterranean region, olive groves (*Olea europaea* subsp. *europaea*) and vineyards (*Vitis vinifera* subsp. *vinifera*). The study area was framed using 1004 square cells, each 10 × 10 km in size. Potential biocontrol services were determined at all those 10 × 10 km grid-cells in which each crop was present as the proportion of the realized out of all potential pairwise interactions between vertebrates and pests. Landscape correlates of biocontrol potential were also explored. Our work suggests that both birds and bats can effectively provide biocontrol services in olive groves and vineyards as they prey many insect pest species associated to both crops. Moreover, it demonstrates that these potential services are impacted by landscape-scale features and that this impact is consistent when evaluated over broad spatial extents. Thus, biocontrol potential by vertebrates significantly increases with increasing amount of natural area, while decreases with increasing area devoted to target crops, particularly olive groves. Overall, our study highlights the suitability of our interaction-based approach to perform spatially-explicit assessments of potential biocontrol services by vertebrates at local spatial scales and suggest its utility for integrating biodiversity and ecosystem services in conservation planning over broad spatial extents.

## Introduction

Landscape modification for agricultural purposes is widely recognized as being one of the major threats to global terrestrial biodiversity^1^. Typically, as landscape modification increases, native vegetation is progressively lost and land becomes dominated by large homogeneous patches of agricultural land. This landscape-scale modification strongly influences local patterns of species richness and abundance because of the lack of opportunity for spill-over between complementary resources^2^. From the agricultural perspective, this is somewhat puzzling as biodiversity is widely recognized to provide ecosystem services that support both crop yield and quality^3^. One such biodiversity-dependent ecosystem services is biological pest control or biocontrol services, defined as the impact of naturally occurring predators (i.e., biocontrol agents) on the population density of pests. A burgeoning research literature demonstrates the substantial economic value of biocontrol services in production landscapes, particularly those provided by flying vertebrates such as insectivorous birds and bats^4–6^. Biocontrol services are thus not surprisingly calling to enter into regional policy and planning agendas, just like other biodiversity-mediated ecosystem services^7–10^. Yet, we still lack rigorous approaches for evaluating landscape correlates of biocontrol services provided by vertebrates to better inform land-use and management decisions.

**Figure 1:**
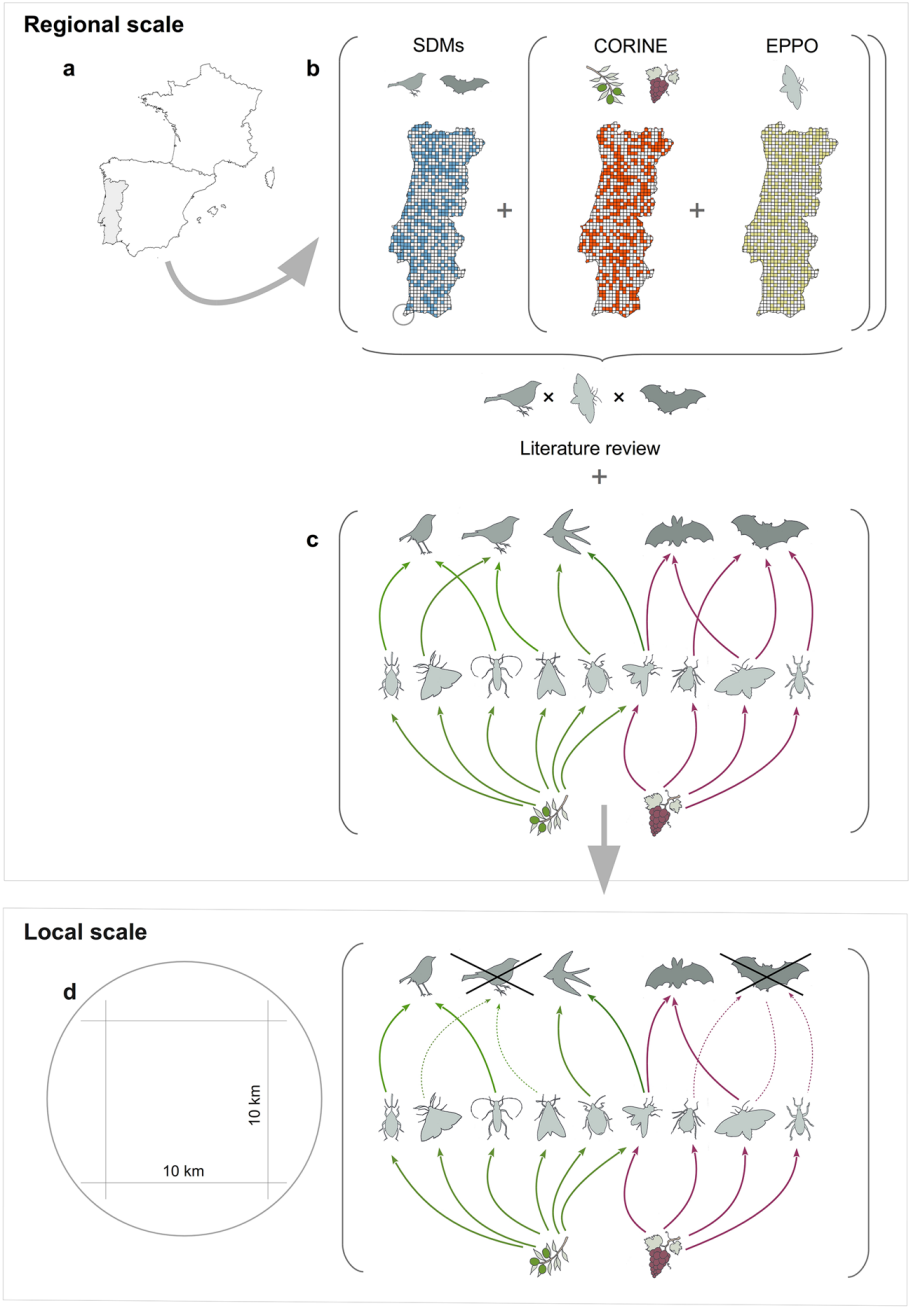
Study region and methodological framework. (**a**) Location of the study area, shaded area (Portugal, Western Iberian peninsula). (**b**) Species distribution models (SDMs) were used to determine the occurrence patterns (presence/absence) of vertebrate species, while data from CORINE Land Cover (CLC) and the European and Mediterranean Plant Protection Organization (EPPO) were used to determine the occurrence patterns of crops and their associated insect pests, respectively. Coloured squares represent the hypothetical distributions of the variables they represent. (**c**) Information on the consumption of pest species by predators was obtained through a comprehensive literature review. (**d**) For every 10 × 10 km grid cell in which crops were present, we calculated a crop-specific biocontrol service index (*bsi*), that represents a proxy for the relative completeness of the interaction assemblage involved in the provision of biocontrol services.

Here, we present results of a spatially-explicit countrywide assessment of the effects of landscape composition on potential biocontrol services provided by birds and bats in two widespread and highly economically valuable Mediterranean crops. Specifically, we evaluate landscape correlates of biocontrol services against insect pests associated with olive groves (*Olea europaea* subsp. *europaea*) and vineyards (*Vitis vinifera* subsp. *vinifera*) in Portugal, western Iberian Peninsula (Fig. 1). First, we performed a comprehensive literature review to identify all vertebrate species that prey on insect pests and, in turn, to determine their potential to act as crop-specific biocontrol agents. Then, we framed the study region using a homogeneous gridding, and modelled the occurrence (presence-absence) patterns of vertebrates throughout the study region using individual grid cells as study unit. Potential biocontrol services were estimated by analysing co-occurrence patterns between vertebrates and insect pests, considering that they reach their maximum when all vertebrates identified as biocontrol agents for a given are present. Finally, landscape-scale correlates of biocontrol services were explored, namely the amount of natural and semi-natural vegetation and that of each individual crop. We hypothesise that, by preying on most insect pests associated with olive groves and vineyards, birds and bats effectively act as biocontrol agents in olive groves and vineyards. We also expect that the potential of biocontrol agents to provide biocontrol services will be impacted by landscape composition and that this impact will be consistent from local to regional extents. Finally, we hypothesise that the impact of landscape composition on biocontrol services will be crop-specific.

## Methods

### Spatial framework and study crop system

The study region was mapped using homogeneous gridding, which was also applied to vertebrate distribution data as well as to potential vertebrate-mediated biocontrol services (see below; Fig. 1). We used 10 × 10 km in size square grids. The entire study region comprised a total of 1004 grids. We focused on olive groves (*Olea europaea* subsp. *europaea*) and vineyards (*Vitis vinifera* subsp. *vinifera*). The reason for selecting these crops was twofold. First, olive groves and vineyards are among the most widespread and economically relevant crops in the Euro-Mediterranean region. Indeed, Portugal is the fourth biggest European producer of both olive oil and grapevine^11^. Second, insects are among the most harmful pests to both crops, causing huge direct costs associated to yield loss and indirect costs related to agrochemicals inputs^12,13^.

Crop-specific occurrence maps (as estimated by the presence-absence of each crop at every individual 10 × 10 grid-cell throughout the study region) were constructed using the CORINE Land Cover (CLC) 2018 database (Supplementary Figure S1). Information about the most harmful insect pests affecting each crop was extracted from EPPO, the European and Mediterranean Plant Protection Organization database^14^. Only insect pests confirmed for mainland Portugal were considered. Unfortunately, Portugal still lacks spatially explicit maps depicting the distribution and incidence of crop pests. This ultimately hampered the use of SDMs to determine landscape correlates behind the distribution and incidence patterns of insects as it was made for vertebrates. As a consequence, the distributions of insect pests were linked to crop occurrence. We acknowledge that at least some pest species can exhibit spatial variations in their distribution and abundance patterns. However, quantitative syntheses of this literature suggest that the abundance of pests often show no significant response to landscape patterns^15^.

### Mapping the distribution of vertebrates

The latest available atlases of birds and bats of mainland Portugal differ in their temporal ranges (1999–2005 and 2010–2012, respectively^16,17^. Thus, to model the current distribution of insectivorous birds and bats we determined species’ past distribution patterns and projected them to recent times using Species Distribution Models (SDMs)^18^. Both bioclimatic and land cover variables at every 10 × 10 km grid-cell were integrated in SDMs to determine the environmental mechanisms underlying species-specific occurrence patterns of vertebrates (Fig. 1). Bioclimatic data was extracted from WorldClim—Global Climate Data^19^. We first considered the complete set of bioclimatic variables (BIO1–BIO19) as well as wind speed (m s^−1^) and solar radiation (kJ m^−2^ day^−1^). However, some of these variables were highly correlated and were discarded when they achieved pairwise correlations *r* > 0.70. Land cover data was extracted from CLC, using CLC 2006 and CLC 2012 to determine past distribution patterns of bird and bat species, respectively, and CLC 2018 (the latest available) to project their current distribution patterns. Due to the high number of land cover types available, these were summarized into broader groups: (1) forests (i.e. natural woodlands, riparian forests and green parks), (2) open agricultural areas (low crops such as cereals and cut forests), (3) tree plantations (pine and eucalyptus afforestations and tree-like crops), (4) urbanized areas (e.g. urban and industrial areas, building grounds), (5) transportation infrastructures (roads, railroad tracks) and (6) water and wetlands (rivers and water bodies). Species-specific habitat suitability maps were then used to build occurrence (i.e. presence-absence) maps for each vertebrate species^20^.

To determine which landscape correlates underlay the occurrence (presence-absence) patterns of vertebrate species, we distinguished between their potential distribution (locations where species could be present) and realized distribution (locations where species are actually present). Potential distribution patterns were determined using only climatic variables, while realized distributions were obtained using both climatic and land cover variables^21^. Differences between occurrence patterns were subsequently used to determine the relative impact that different land cover types (i.e., the amount of natural and semi-natural vegetation and that of each target crop) has on the occurrence of every vertebrate species. We used Generalized Linear Models (GLMs) to develop potential-realized species-specific distributions as well as to determine the landscape correlates underlying their species-specific occurrence patterns. All models were evaluated using a repeated random cross-validation, a resampling approach to assess the robustness of predictions whereby we randomly resampled 2/3 of the original data, calculated the parameter estimates on the best model, and used them to obtain predicted values for the remaining 1/3 of the dataset^20^. For each vertebrate species, we calculated the average and standard error of AUC based on 100 realizations of the above described cross-validation procedure. We only retained species-specific models providing robust estimates (i.e. AUC values ≥ 0.7). Thus, from a total of 72 insectivorous bird and 24 bat species which were initially included into SDMs, 66 birds (91.6%) and 12 bats (50.0%) were retained for further analyses (Supplementary Figure S2).

### Linking vertebrates, crops and pests

Vertebrates are linked to crops through the trophic relationship they maintain with crop-specific insect pests. We thus constructed a tri-trophic interaction matrix between vertebrates, crops and pests. We first constructed the interaction matrix between pests and crops using the EPPO database. This interaction matrix was then joined with an interaction matrix relating vertebrates and pests, ultimately enabling the establishment of a link between vertebrates and crops and, in turn, determining the potential role of the vertebrates to act as biocontrol agents for each crop. The interaction matrix between vertebrates and pests was constructed by performing a comprehensive literature review aimed at collecting information regarding the consumption of crop-specific pests by vertebrates. We searched in the Web of Knowledge and Google Scholar combining the following keywords for vertebrates (‘bird*’ OR ‘bat*’ OR ‘vertebrate*’ OR ‘predator’), pests (‘insect’ OR ‘invertebrate*’ OR ‘arthropod’ OR ‘pest’ OR “prey”) and processes (‘diet’ OR ‘prey selection’ OR ‘biocontrol’ OR ‘pest control*’). We additionally searched for citations in general books potentially providing information about dietary composition of either birds or bats. Studies where the interaction between predators and pests was inferred rather than measured were excluded. A total 112 studies was reviewed (Supplementary Table S1).

### Mapping and evaluating crop-specific biocontrol services

We mapped crop-specific biocontrol services using the pairwise interactions between vertebrates and insect pests^22^. Because no previous information on the relative impact of each vertebrate species on pests is available, we considered pairwise interactions between vertebrates and pests as being redundant (sensu^23^). In other words, the contributions of vertebrate species to biocontrol services were considered to be equivalent and replaceable. We thus considered that biocontrol services reached their maximum in a given cell when all the interactions between vertebrates and pests are present as estimated from the realized-potential distributions of vertebrates. From this perspective, considering *m* the total number of pests associated with olive groves (*m* = 17) and vineyards (*m* = 38), the relative contribution of a pairwise interaction (*w*_*j*_) between a given vertebrate (*v*) and pest (*p*) to biocontrol services was calculated as:

1 $$ {w_{j} = \frac{{1}}{{\sum\nolimits_{i} {v_{i} } \cdot p_{j} }}}\quad {\text{for}}\quad j = {1},{2}, \ldots ,m $$

where ${v_{i} \cdot p_{j} }$ is the interaction between the vertebrate *i* and the pest *j*. For instance, if pest *j* is consumed exclusively by one vertebrate species (either bird or bat), then *w*_*j*_ = 1, if it is consumed by two vertebrate species, then *w*_*j*_ = 0.5, and so forth. In other words, *w*_*j*_ index weights each (unidirectional) interaction based on the contribution of a given vertebrate species when preying on a given pest species. For each crop we thus defined a simple predation pressure (*P*) index based on the number of existing interactions between vertebrates and pests. The *P* index was defined as:

2 $$ {P = \sum\limits_{j} {\sum\limits_{i} {\left( {v_{i} \cdot p_{j} \cdot w_{j} } \right)} } } $$

Finally, a crop-specific biocontrol service index (*bsi*) was calculated as:

3 $$ {bsi = \frac{{P_{real} }}{{P_{pot} }}} $$

where *P*_*real*_ is the predation pressure exerted by all those vertebrates estimated to be present based on their species-specific realized distribution and *P*_*pot*_ is the predation pressure exerted by all those vertebrates estimated to be present based on their species-specific potential distribution. The *bsi* index therefore indicates the relative completeness of the interaction assemblage involved in the provision of biocontrol services for each crop, ranging between 0 (minimum) and 1 (maximum).

### Determining landscape-scale correlates of vertebrate-mediated biocontrol services

We were interested in determining the environmental correlates of biocontrol services (as estimated by *bsi*). Crop-specific *bsi* values were thus correlated with both the amount of natural and semi-natural areas as well as with the amount of each target crop to determine how landscape composition affects the relative completeness of the interaction assemblage. On one hand, natural and semi-natural vegetation integrated the categories 3.1 (Forests) and 3.2 (Scrub and/or herbaceous vegetation) belonging to the Level 2 of the CLC 2018 database and the categories 2.4.3 (Land occupied by agriculture, with significant areas of natural vegetation) and 2.4.4 (Agro-forestry areas) belonging to the Level 3 of the CLC database. On the other hand, olive grove and vineyard cover was extracted from the CLC database, namely from the 2.2.3 and 2.2.1 Level 3 categories, respectively. Olive groves and vineyards integrated the group (3) of variables used to model vertebrate occurrence patterns (see above).

To tackle the presence of spatial autocorrelation found in exploratory Generalized Linear regression Models, we used Spatial Autoregressive Models (SARs) with the queen contiguity, which considers that two grid cells are considered contiguous if they share a common border or vertex. The spatial weights matrix for contiguous grid cells was obtained by row standardisation (commonly known as style *W,* considered here as the centroid of the 10 × 10 km cell). This models are particularly suitable to describe the relationship between independent and dependent variables by involving location effect of the data. In our case, we used SARs models to model the *bsi* values for both olive groves and vineyards (response or dependent variables) against the amount of natural and semi-natural area and that of each target crop (explanatory or independent variables). Response variables were log transformed.

No sign of collinearity between explanatory variables was found for both models (VIF values < 2). The assumptions of homocedasticity and independence of the data points were validated for both the olive groves and the vineyards models by the Breusch-Pagan test (p values of 0.063 and 0.071, respectively) and Moran’s I test (p values of 0.687 and 0.397, respectively), on the model residuals.

## Results

**Figure 2:**
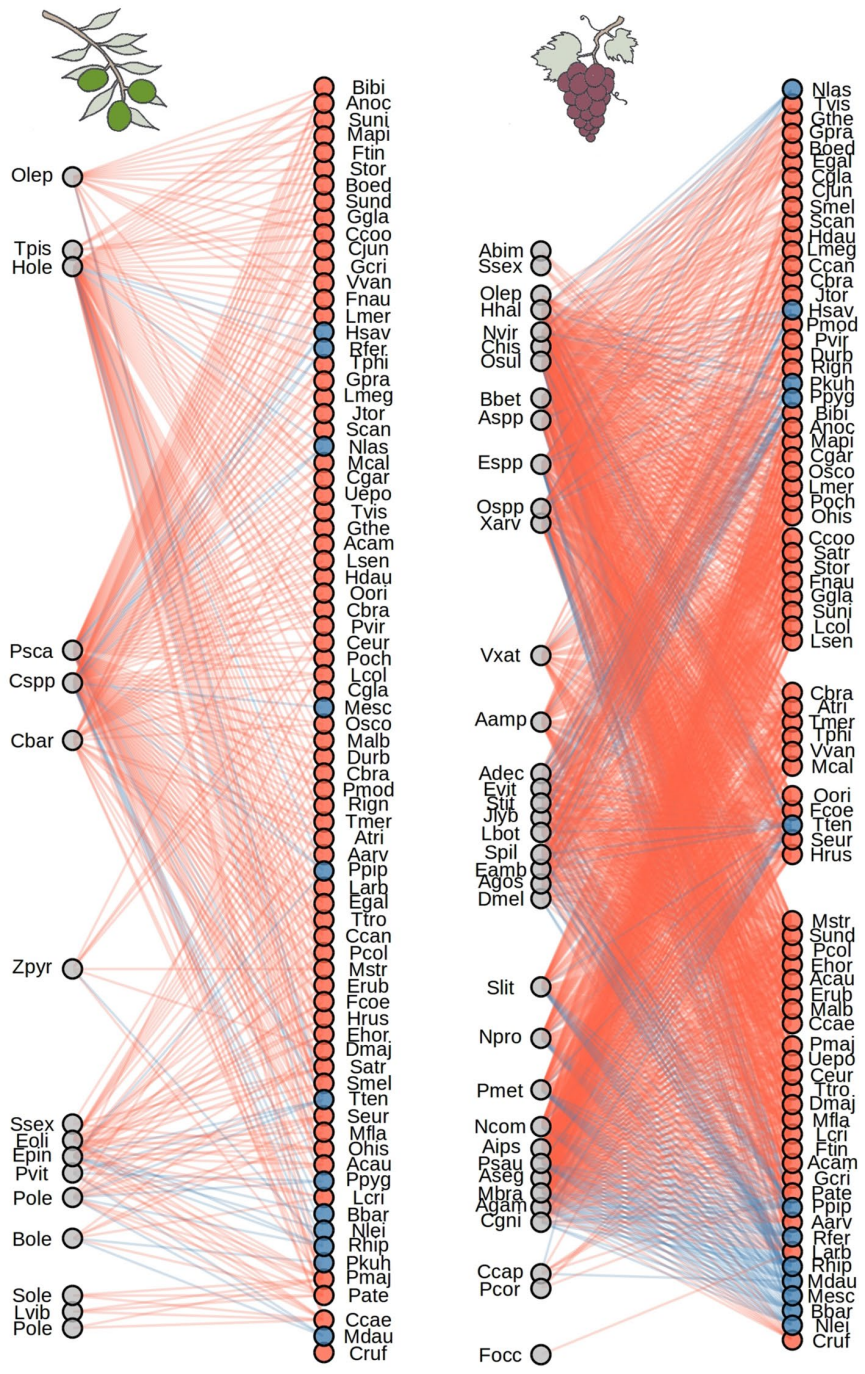
Trophic interaction links between vertebrates and olive (left panel) and grape (right panel) pests. Grey circles represent pest species of each crop, whereas red and blue circles represent bird and bat species, respectively. Trophic interactions between pests and vertebrates were obtained through a literature review (Supplementary Table S1). Identities of both vertebrate and pest species were simplified by using four-letter abbreviations.

A total of 1,853 pairwise interactions between vertebrates (*n* = 78) and insect pests (*n* = 50) were identified (Fig. 2). While every vertebrate species was found to prey on at least one pest associated with olives and grapes (Fig. 2), on average olive pests were preyed by a lower number of vertebrates than grape pests (22.59 ± 6.03, range: 2–69 and 41.68 ± 3.68, range: 1–69, respectively). Similarly, on average, vertebrates preyed on a higher number of grape pests (19.88 ± 0.91, range: 4–33) than olive pests (4.86 ± 0.27, range: 1–12) (Fig. 2).

**Figure 3:**
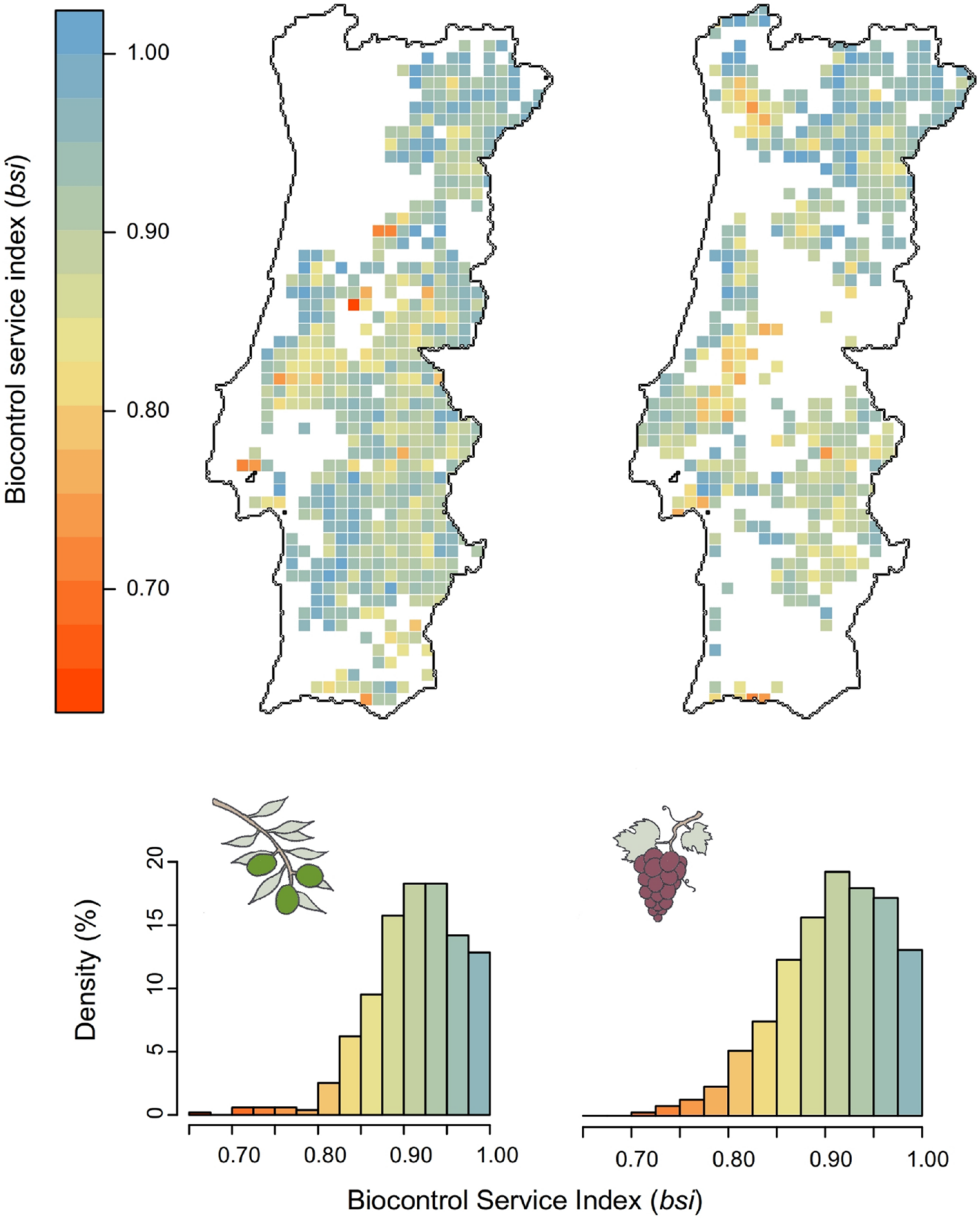
Biocontrol service indices (*bsi*) in olive groves (left panels) and vineyards (right panels) throughout Portugal. Upper panels show the spatial distribution patterns of estimated *bsi* values for each crop, while lower panels depict the frequency distribution histograms (%) using the 10 × 10 km grid cells as unit. Maps were generated using the free and open source Geographic Information System QGIS v2.8 (https://qgis.org/en/site/).

**Figure 4:**
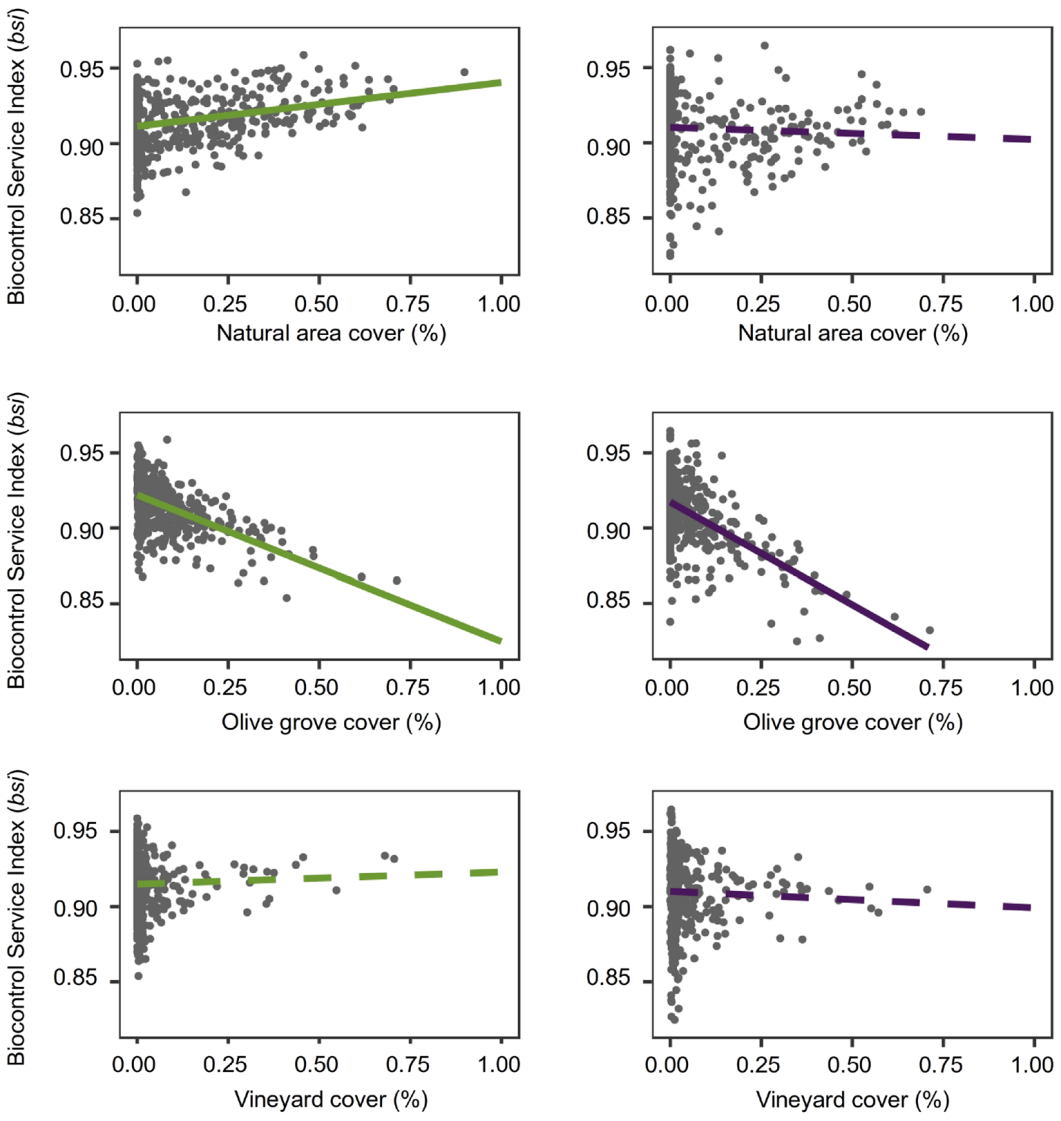
Linear relationships between biocontrol services indices (*bsi*) and landscape composition (proportions of natural area and that of each crop at 10 × 10 km grid cells) estimated for olive groves (left panels) and vineyards (right panels). Significant relationships between *bsi* and landscape composition are shown using continuous lines, while non-significant relationships are shown using dashed lines (see Table 1).

Average *bsi* values were relatively high and quite similar between vineyards and olive groves (0.909 ± 0.003 and 0.914 ± 0.002, respectively). However, the lower limit of the range were slightly lower in olive groves than in vineyards (Fig. 3). The amount of natural and semi-natural vegetation significantly influenced *bsi* values in olive groves, but not in vineyards (Table 1). Thus, the higher the amount of natural and semi-natural areas in a given grid-cell, the higher the *bsi* value estimated for olive groves in such a cell (Fig. 4). Remarkably, the amount of olive groves significantly influenced *bsi* values estimated for both crops. The higher the area covered by olive groves in a given grid cell, the lower the *bsi* value estimated for both olive groves and vineyards (Fig. 4). We found no influence of the proportion of vineyards on the *bsi* values estimated for vineyards or olive groves (Table 1).

**Table 1:** Results of the spatial autoregressive model (SAR) relating the proportion of the three land-cover types on the Biocontrol Service Index (*bsi*) estimated for all those 10 × 10 grid cells in which olive groves and vineyards were present.

	Estimate	SE	*p* value	R^2^
**Olive grove (*bsi*) (*n* = 514)**				
Spatial lag (rho)	0.445	0.054	** < 0.001**	15.5%
Intercept	− 0.048	0.006	** < 0.001**	
Natural area cover	0.026	0.013	**0.030**	
Olive grove cover	− 0.076	0.024	**0.002**	
Vineyard cover	− 0.006	0.029	0.500	
**Vineyard (*bsi*) (*n* = 439)**				
Spatial lag (rho)	0.496	0.049	** < 0.000**	22.3%
Intercept	− 0.043	0.006	** < 0.000**	
Natural area cover	0.015	0.018	0.398	
Olive grove cover	− 0.102	0.028	** < 0.000**	
Vineyard cover	− 0.023	0.029	< 0.428	

Significant *p* values (*p* < 0.01) are highlighted in bold.

## Discussion

In close agreement with previous literature research, our interaction-based approach demonstrates that landscape composition impacts the potential of birds and bats to provide biocontrol services in agroecosystems^24–28^. Moreover, we found that this landscape-level impact is crop-specific. Thus, while potential biological services against olive pests increases with natural vegetation cover and decreases with olive grove cover, potential biocontrol services against grape pests is only (negatively) related to olive grove cover. We hypothesise that the idiosyncratic spatial structure of olive groves and vineyards may be well behind this response pattern.

On one hand, the latest results of the five-yearly European Union orchard survey indicate that olive grove cover in Portugal accounts for about 400.000 hectares, thereby representing a significant share of the land surface devoted to agriculture in this country^11^. Portugal is indeed the fourth most important olive producing country in Europe, only overtaken by Spain, Italy and Greece. Even more important is the fact that olive groves typically cover broad spatial extents, driving a strong structural simplification of the landscapes in which they are embedded. As reported for other crops across the world, the structural simplification of olive-growing landscapes is known to severely impact species richness and abundance of vertebrates (including birds^29–31^ and bats^32^, with potential concomitant effects on biocontrol services^33,34^). Indeed, existing knowledge suggests that large and homogeneous olive monocultures are far from being true foraging habitats for birds and bats, except for some avian guilds such as wintering frugivores^35^. Thus, the significant share that olive groves represents may well explain the negative influence they exert on potential biological services provided by vertebrates against olive pests, but also against pests associated with any adjacent crop including vineyards.

On the other hand, while vineyards are also widespread in Portugal, the area covered by this crop is about 200.000 hectares, i.e., half of that covered by olive groves. Moreover, unlike olive groves, vineyards rarely cover large spatial extents as monocultures, but they are typically embedded in landscape mosaics. Such is the case, that landscape features are suggested to be a more important correlate of vertebrate richness and activity in vineyards –and consequently of potential biocontrol services– than the characteristics of vineyards themselves (e.g^26–28,36^). Thus, for example, Pithon et al. found that only a small fraction of the bird species they recorded at the landscape scale were frequent users of vineyards plots, and that most species selected adjacent land cover types^37^. Moreover, Froidevaux et al.^38^ found that even organic farming was ineffective on its own to enhance bat activity and species richness regardless of the landscape context around vineyard plots. Overall, this may explain why we found that the area occupied by vineyards had no influence on potential biological services against grape pests, but why it was significantly influenced by other co-occurring land-cover types, particularly by the widespread olive groves.

Despite our results strongly agree with previous studies, we acknowledge that the impact of landscape composition on potential biocontrol services potential is to a certain extent weaker than expected. This is because landscape features around olive groves and vineyards have been found to exert a strong (and deleterious) impact on the occurrence probabilities of flying vertebrates, including both birds and bats (see references above). We hypothesise that the scale of our spatial approach (i.e., 10 × 10 km) is behind this response pattern. It should be noted that most previous studies used smaller spatial scales to determine the influence of landscape composition on vertebrate occurrence patterns and potential biocontrol services within olive groves and vineyard plots. Reasonably, this is made to better cope with the foraging range of most species and, in turn, to better understand the effects of landscape composition on occurrence patterns at both species and community level^18^. However, conservation planning at regional extents needs to reach a compromise between the spatial scale at which ecological processes operate and the spatial scale at which territories are managed^39^. In this way, our results demonstrate the suitability of our spatial framework to inform land-use and management decisions and underline their utility for integrating biodiversity and ecosystem services into conservation planning over broad spatial extents.

### Caveats and the way forward

Due to the lack of rigorous spatially-explicit data on the distribution of both vertebrates and insect pests, our interaction-based approach to potential biocontrol services is based on their co-occurrence probabilities. While species loss inevitably causes the loss of interactions in which they are involved, many species can be present at such low densities that they can be considered virtually extinct from an ecological point of view^40^. It is important to highlight this because previous studies suggest that landscape composition may have even stronger effects on vertebrate species abundance than on species occurrence than those reported here^41^. Moreover, similarly, despite the occurrence patterns of insect pests generally overlap those of their target crops, their relative incidence exhibit strong geographical variations^42^. This being the case, we encourage governmental institutions to develop updated and rigorous distribution maps of both vertebrates and insect pests to better understand the effects of landscape composition on their distribution patterns and, in turn, to better predict the vulnerability of biocontrol services to landscape modification.

Another key challenge of our approach is related to the fact that we consider all pairwise interactions between vertebrates and pests as being equivalent^22^. We acknowledge this assumption may not be necessarily well warranted as it implies that all vertebrates are equivalent in terms of the quality of their species-specific interactions with pests. Once again, this theoretical commitment was taken due to the lack of detailed data regarding the dietary niche of vertebrates and, in turn, their relative potential as biocontrol agents against insect pests. Fortunately, a burgeoning research literature is shedding light on this knowledge gap thanks to advanced molecular biology techniques such as DNA metabarcoding (e.g.^43^). Thus, the dietary analysis of a rapidly increasing number of vertebrates is being performed, remarkably to identify their role as biocontrol agents of crops pests^28^. By the way, these dietary analysis will also help to better understand the trophic relationship between insectivorous vertebrates and insect pests and, in turn, the net effect of vertebrates on potential biocontrol services. This is because, ultimately, these dietary analysis will help to disentangle the direct but also indirect effects of vertebrates on pests by accounting for the potential impact that vertebrates has on predatory species belonging to lower trophic levels^44^. However, both the high number of vertebrate and pest species as well as the strong geographical variations in resource use exhibited by vertebrates will considerably delay the fulfilment of this challenging task^45^.

In sum, our study highlights the suitability of interaction-based approaches to perform spatially-explicit assessments of potential vertebrate-mediated biocontrol services, and underlines their relevance for broad-scale conservation planning. Though we focused on olive groves and vineyards, we suggest that our approach can be applied to other cropping systems involving insect pests. Moreover, we encourage the assessment of the suitability of this approach for other interaction-based ecosystem services, such as crop pollination.

The potential of vertebrates to provide biocontrol services in olive groves and vineyards were impacted in homogeneous olive-growing landscapes. Because current trends predict that the land surface devoted to this crop will continue to increase in coming years, our approach and main findings are highly relevant for the conservation management planning of agricultural landscapes using biocontrol services as incentives for biodiversity maintenance.

## Supplementary Information

Supplementary Information.

## References

[1] Foley JA (2005). Global consequences of land use. Science.

[2] Fischer J, Lindenmayer D (2005). Landscape modification and habitat fragmentation: a synthesis. Global Ecol. Biogepogr..

[3] Dainese M (2019). A global synthesis reveals biodiversity-mediated benefits for crop production. Sci. Adv..

[4] Boyles J, Cryan P, McCracken GF, Kunz TH (2011). Economic importance of bats in agriculture. Science.

[5] Puig-Montserrat X (2015). Pest control service provided by bats in Mediterranean rice paddies: linking agroecosystems structure to ecological functions. Mamm. Biol..

[6] Maas B (2015). Bird and bat predation services in tropical forests and agroforestry landscapes. Biol. Rev..

[7] Maes J (2012). Mapping ecosystem services for policy support and decision making in the European Union. Ecosyst. Serv..

[8] Alkemade R, Burkhard B, Crossman ND, Nedkov S, Petz K (2014). Quantifying ecosystem services and indicators for science, policy and practice. Ecol. Indic..

[9] Mandle L (2017). Assessing ecosystem service provision under climate change to support conservation and development planning in Myanmar. PLoS ONE.

[10] Dang AN, Jackson BM, Benavidez R, Tomscha SA (2021). Review of ecosystem service assessments: Pathways for policy integration in Southeast Asia. Ecosyst. Serv..

[11] Eurostats. Agriculture, Forestry and Fisheries. European Statistics. https://ec.europa.eu/eurostat (2021).

[12] Eurostats. Pests and diseases in viticulture. EIP-AGRI Focus Group. https://ec.europa.eu/eip/agriculture/ (2019).

[13] Eurostats. Pests and diseases of the olive tree. EIP-AGRI Focus Group. https://ec.europa.eu/eip/agriculture/ (2019).

[14] EPPO. EPPO Global Database. https://gd.eppo.int (2018).

[15] Chaplin-Kramer R, O'Rourke ME, Blitzer LJ, Kremen C (2011). A meta-analysis of crop pest and natural enemy response to landscape complexity. Ecol. Lett..

[16] Equipa Atlas. Atlas das Aves Nidificantes em Portugal (1999–2005). Instituto da Conservação da Natureza e da Biodiversidade, Sociedade Portuguesa para o Estudo das Aves, Parque Natural da Madeira e Secretaria Regional do Ambiente e do Mar. Assírio & Alvim, Lisboa (2008).

[17] Rainho, A., Alves, P., Amorim, F. & Marques, J. T. Atlas dos morcegos: de Portugal continental. Instituto da Conservação da Natureza e das Florestas (2013).

[18] Herrera JM, Ploquin E, Rodriguez-Pérez J, Obeso JR (2014). Determining habitat suitability of a mountain bumblebee fauna: a baseline approach for testing the impact of climate change on species distribution and abundance. J. Biogeogr..

[19] Hijmans RJ, Cameron SE, Parra JL, Jones PG, Jarvis A (2005). Very high resolution interpolated climate surfaces for global land areas. Int. J. Climatol..

[20] Araújo MB (2019). Standards for distribution models in biodiversity assessments. Sci. Adv..

[21] Jiménez-Valverde A, Lobo JM, Hortal J (2008). Not as good as they seem: the importance of concepts in species distribution modelling. Divers. Distrib..

[22] Tylianakis JM, Laliberté E, Nielsen A, Bascompte J (2010). Conservation of species interaction networks. Biol. Conserv..

[23] Valiente-Banuet A (2015). Beyond species loss: the extinction of ecological interactions in a changing world. Funct. Ecol..

[24] Karp DS (2013). Forest bolsters bird abundance, pest control, and coffee yield. Ecol. Lett..

[25] Maas B, Clough Y, Tscharntke T (2013). Bats and birds increase crop yield in tropical agroforestry landscapes. Ecol. Lett..

[26] Barbaro L (2016). Avian pest control in vineyards is driven by interactions between bird functional diversity and landscape heterogeneity. J. App. Ecol..

[27] Paiola A (2020). Exploring the potential of vineyards for biodiversity conservation and delivery of biodiversity-mediated ecosystem services: a global-scale systematic review. Sci. Total Environ..

[28] Charbonnier Y (2021). Pest control services provided by bats in vineyard landscapes. Agric. Ecosyst. Environ..

[29] Rey PJ (2020). Landscape-moderated biodiversity effects of ground herb cover in olive groves: implications for regional biodiversity conservation. Agr. Ecosyst. Environ..

[30] Morgado R (2020). A Mediterranean silent spring? The effects of olive farming intensification on breeding bird communities. Agric. Ecosyst. Environ..

[31] Martínez-Núñez C (2020). Direct and indirect effects of agricultural practices, landscape complexity and climate on insectivorous birds, pest abundance and damage in olive groves. Agric. Ecosyst. Environ..

[32] Herrera JM, Costa P, Medinas D, Marques JT, Mira A (2015). Community composition and activity of insectivorous bats in Mediterranean olive farms. Anim. Conserv..

[33] Costa A (2020). Structural simplification compromises the potential of common insectivorous bats to provide biocontrol services against the major olive pest *Prays oleae*. Agric. Ecosyst. Environ..

[34] Puig-Montserrat X, Mas M, Flaquer C, Tuneu-Corrala C, López-Baucells A (2021). Benefits of organic olive farming for the conservation of gleaning bats. Agric. Ecosyst. Environ..

[35] Rey PJ (2011). Preserving frugivorous birds in agro-ecosystems: lessons from Spanish olive orchards. J. Appl. Ecol..

[36] Rodríguez-San Pedro A (2018). Influence of agricultural management on bat activity and species richness in vineyards of central Chile. J. Mamm..

[37] Pithon JA, Beaujouan V, Daniel H, Pain G, Vallet J (2016). Are vineyards important habitats for birds at local or landscape scales?. Basic Appl. Ecol..

[38] Froidevaux JSP, Louboutin B, Jones G (2017). Does organic farming enhance biodiversity in Mediterranean vineyards? A case study with bats and arachnids. Agr. Ecosyst. Environ..

[39] Van der Biest K (2020). Aligning biodiversity conservation and ecosystem services in spatial planning: focus on ecosystem processes. Sci. Total Environ..

[40] Janzen DH (2001). Latent extinction-the living dead. Encycl. Biodivers..

[41] Herrera JM (2016). Generalities of vertebrate responses to landscape composition and configuration gradients in a highly heterogeneous Mediterranean region. J. Biogeogr..

[42] Ponti L, Gutierrez AP, Rutid PM, Dell’Aquila A (2014). Fine-scale ecological and economic assessment of climate change on olive in the Mediterranean Basin reveals winners and losers. Proc. Nat. Acad. Sci..

[43] Silva LP (2019). Advancing the integration of multi-marker metabarcoding data in dietary analysis of trophic generalists. Mol. Ecol. Resour..

[44] Pejchar L (2018). Net effects of birds in agroecosystems. Bioscience.

[45] Alberdi A (2020). DNA metabarcoding and spatial modelling link diet diversification with distribution homogeneity in European bats. Nat. Comm..

