# Supplementary material for: A food web approach reveals the vulnerability of biocontrol services by birds and bats to landscape modification at regional scale
Source: Sci Rep. 2021 Dec 8;11:23662. doi: 10.1038/s41598-021-02768-0 (PMC8654945; doi:10.1038/s41598-021-02768-0)
Supplement: Supplementary file 1 — Supplementary Information. [file 41598_2021_2768_MOESM1_ESM.doc]

**Supplementary Information**

A food web approach reveals the vulnerability of biocontrol services by birds and bats to landscape modification at regional scale

José M. Herrera^1*^, Bruno Silva^1^, Gerardo Jiménez-Navarro^1^, Silvia Barreiro^1^, Nereida Melguizo-Ruiz^1^, Francisco Moreira^2,3^, Sasha Vasconcelos^2,3^, Rui Morgado^2,3^ & Javier Rodriguez-Pérez^1,4^

^1^Mediterranean Institute for Agriculture, Environment and Development and University of Évora, Casa Cordovil, 2nd Floor, R. Dom Augusto Eduardo Nunes 7, 7000 – 651 Évora (Portugal)

^2^Centro de Ecologia Aplicada “Professor Baeta Neves” (CEABN), InBIO, Instituto Superior de Agronomia and Universidade de Lisboa, Tapada da Ajuda, 1349 – 017 Lisboa, (Portugal)

^3^Centro de Investigação em Biodiversidade e Recursos Genéticos (CIBIO), InBIO and Universidade do Porto, Campus Agrário de Vairão, 4485 – 601 Vairão (Portugal)^4^Institute for Multidisciplinary Research in Applied Biology (IMAB), Depto. Ciencias del Medio Natural, Centro Jerónimo de Ayanz, Universidad Pública de Navarra (UPNA), Campus Arrosadía, 31006 Pamplona (Spain)

^*^Corresponding author

E-mail: jmherrera@uevora.pt (JMH)

**Supplementary figure S2.** Maps depicting the proportion of olive groves (left panel) and vineyards (right panel) within each 10 × 10km grid-cells throughout Portugal.


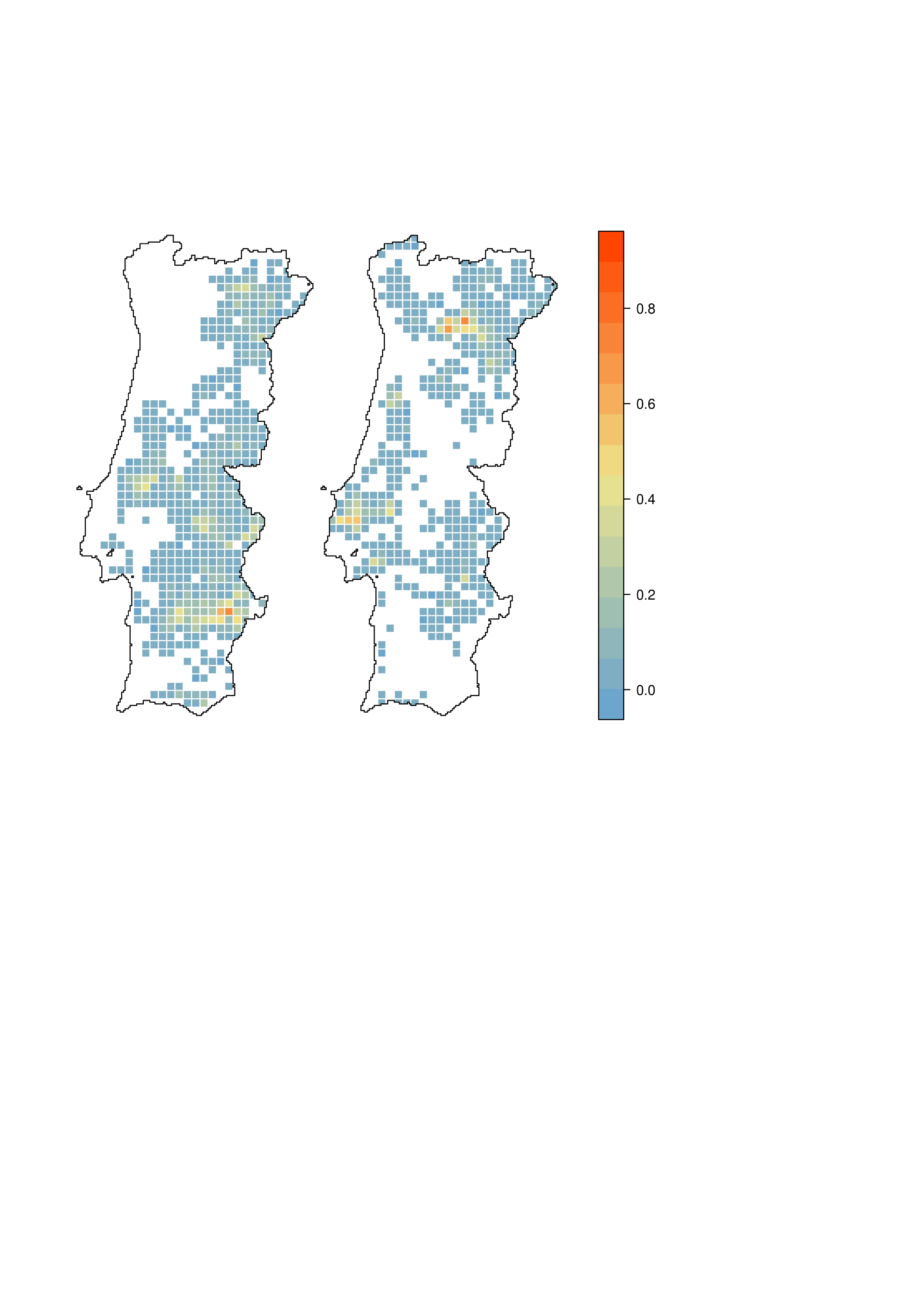


**Supplementary figure S1.** Maps depicting the number (species richness) of birds (up panels) and bats (down panels) at 10 × 10km grid-cells throughout Portugal. Each map is obtained through Species Distribution Models (SDMs) as the sum of species in their potential (right panels) and realized (mid panels) distributions. Left panels represent the difference between potential and realized distributions, thereby the absence of species even when they are in climatically suitable grid cells.

**
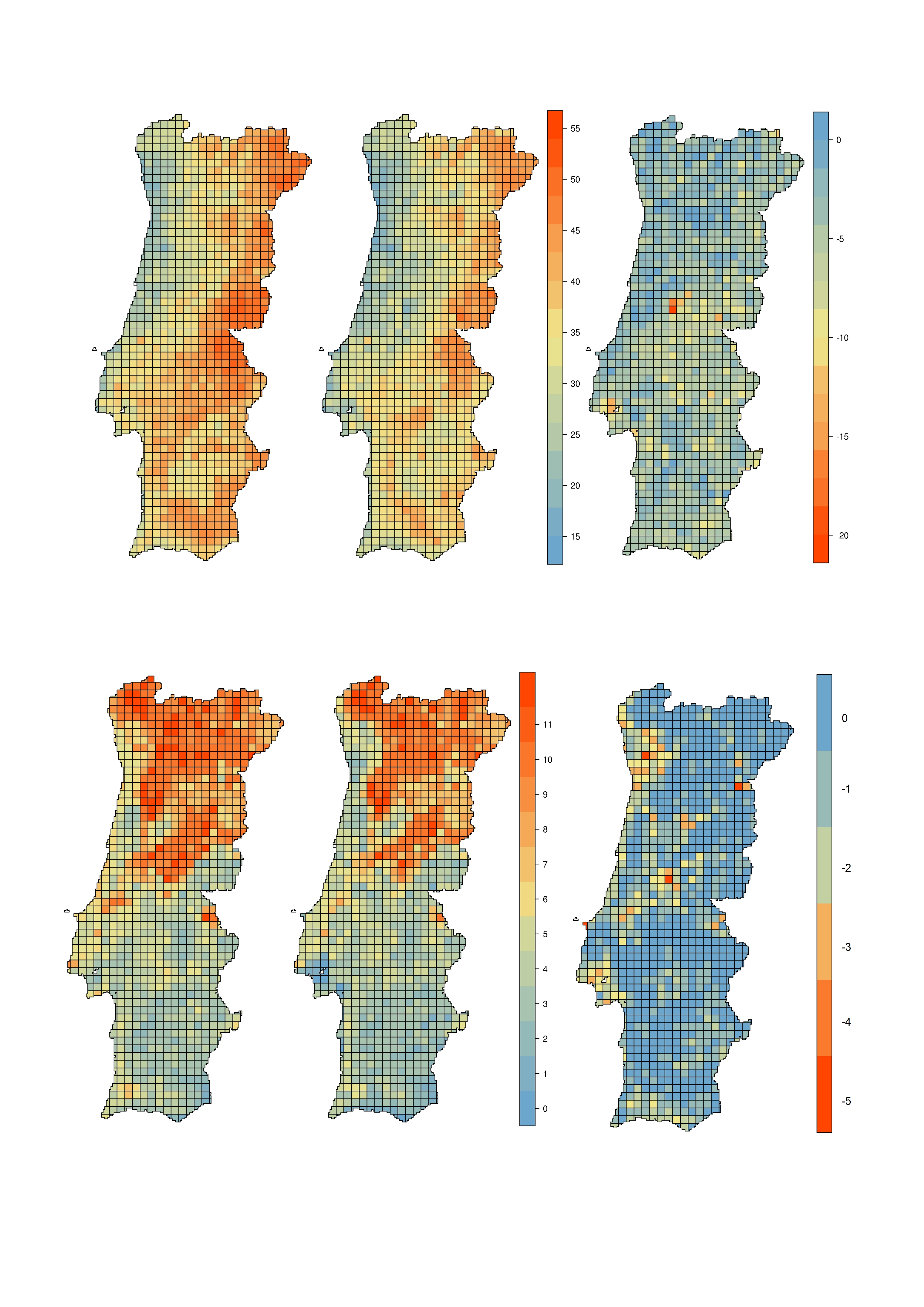
**

**Supplementary Table S1**. List of references used to determine the consumption of crop pests by vertebrates.

| **RefID** | **Reference** |
| --- | --- |
| 1 | Bossema I. (1979). Jays and oaks: an eco-ethological study of a symbiosis. Behaviour, 70: 1-117. |
| 2 | Rolando A. (1998). Factors affecting movements and home ranges in the jay (Garrulus glandarius). Journal of Zoology, 246(3): 249-257. |
| 3 | Mienis H.K., Rittner O., & Vaisman S. (2016). Information concerning Eobania vermiculata, I. On the presence of this exotic species in Israel (Mollusca, Gastropoda, Helicidae). Triton, 34: 29-36. |
| 4 | Sosnowski J., & Chmielewski S. (1996). Breeding biology of the roller Coracias garrulus in Puszcza Pilicka forest (Central Poland). Acta Ornithologica, 31 (2): 119-131 |
| 5 | Cassola F., & Lovari S. (1979). Food habits of Rollers during the nesting season. Bolletino di Zoologia, 46 (1-2): 87-89. |
| 6 | Cónsul C., & Álvarez F. (1978). Dieta alimenticia del Rabilargo (Cyanopica cyanea). Doñana Acta Vertebrata, 5: 73-88. |
| 7 | Canário F., Boieiro M., & Vicente L. (2002). The nestling diet of the Iberian azure-winged magpie Cyanopica cyanus cooki in southeastern Portugal. Ardeola, 49 (2): 283-286. |
| 8 | Milwright R.D.P. (1998). Breeding biology of the Golden Oriole Oriolus oriolus in the fenland basin of eastern Britain. Bird Study, 45 (3): 320-330. |
| 9 | Jackson H.D. & Oatley T.B. (2000). . The food of the Afrotropical nightjars. Journal of African Ornithology, 71: 404-407 |
| 10 | Sáez-Gómez P. & Camacho C. (2016). Chotacabras cuellirrojo – Caprimulgus ruficollis. En: Enciclopedia Virtual de los Vertebrados Españoles. Salvador, A., Morales, M. B. (Eds.). Museo Nacional de Ciencias Naturales, Madrid. http://www.vertebradosibericos.org/ |
| 11 | Jackson H.D., & Oatley T. B. (2000). Food of the nightjars in Zimbabwe. Ostrich, 71(3-4), 404-407. |
| 12 | Orlowski G. & Karg J. (2013). Diet breadth and overlap in three sympatric aerial insectivorous birds at the same location. Bird Study, 60(4), 475-483. |
| 13 | Orłowski G., Karg J., & Karg G. (2014). Functional invertebrate prey groups reflect dietary responses to phenology and farming activity and pest control services in three sympatric species of aerially foraging insectivorous birds. PloS one, 9(12), e114906. |
| 14 | Orłowski G., & Karg J. (2011). Diet of nestling Barn Swallows Hirundo rustica in rural areas of Poland. Central European J. of Biology, 6(6): 1023-1035. |
| 15 | Chisamera G. & Traian M. (2006). Contributions to the knowledge of the food structure of Red-rumped Swallow (Hirundo daurica rufula Temm. 1835)(Passeriformes: Hirundinidae) in Romania and Turkey [Partial results of “Focida” 2006 expedition in Turkey]. ravaux du Muséum National d’Histoire Naturelle “Grigore Antipa, 50, 463-477. |
| 16 | Newton I. (1967). The adaptive radiation and feeding ecology of some British finches. Ibis, 109: 33–98. |
| 17 | Brickle N.W., & Harper D.G.C. (1999). Diet of nestling corn buntings Miliaria calandra in southern England examined by compositional analysis of faeces. Bird Study, 46: 319-329. |
| 18 | Martínez-Cabello A., Soler M., & Soler J.J. (1991). Alimentación del acentor común (Prunella modularis) durante su invernada en el sureste de la Península Ibérica. Ardeola, 38 (2): 305-315. |
| 19 | Bishton G. (1985). The diet and foraging behaviour of the Dunnock Prunella modularis in a hedgerow habitat. Ibis, 128: 526-539. |
| 20 | Gil-Lletget A. (1928). Estudios pobre la alimentación de las aves. Bol. De la R. Soc. Esp. De Hist. Natural, 28: 171-194 |
| 21 | Orlowski G., Karg J., & Czarnecka J. (2011). Orłowski, G., Karg, J., & Czarnecka, J. (2011). Frugivory and size variation of animal prey in Black Redstart Phoenicurus ochruros during summer and autumn in south-western Poland. Ornis Fennica, 88: 161-171 |
| 22 | Hódar J.A. (1998). Individual diet variations in a wintering population of black redstart Phoenicurus ochruros: relationships with bird morphology and food availability. Revue d'Écologie (Terre Vie), 53: 1-77. |
| 23 | Krištín A., & Exnevorá A. (1994). On the diet and breeding biology of Tree Pipit (Anthus trivialis) and Black Redstart (Phoenicurus ochruros). Sylvia, 30: 64-71. |
| 24 | Davies N.B. (1976). Food, flocking and territorial behaviour of the pied wagtail (Motacilla alba yarrellii Gould) in winter. Journal of Animal Ecology, 45 (19): 235-253. |
| 25 | Davies N.B. (1977). Prey selection and social behaviour in wagtails (Aves: Motacillidae).ournal of Animal Ecology, 46: 37-57. |
| 26 | Barba E., Gil-Delgado J.A., & López J.A. (1989). La alimentación de los pollos del carbonero común (Parus major) en el naranjal valenciano. Ardeola, 36: 83-87 |
| 27 | Barba E., & Gil-Delgado J.A. (1990). Seasonal variation in nestling diet of the great tit Parus major in orange groves in eastern Spain. Ornis Scandinavica, 21 (4): 296-298. |
| 28 | Nour N., Currie D., Matthysen E., Van Damme R., & Dhondt A.A. (1998). Effects of habitat fragmentation on provisioning rates, diet and breeding success in two species of tit (great tit and blue tit). Oecologia, 114(4): 522-530. |
| 29 | García-Navas V., Ferrer E.S., Sanz J.J. (2013). Prey choice, provisioning behaviour, and effects of early nutrition on nestling phenotype of titmice. Écoscience, 20 (1): 9-18. |
| 30 | Gibb J.A., & Betts M.M (1963). Food and food supply of nestling tits (Paridae) in Breckland pine. Journal of Animal Ecology, 32 (3): 489-533. |
| 31 | Betts M.M. (1955). The food of titmice in oak woodlan. Journal of Animal Ecology, 24 (2): 282-323 |
| 32 | Solomon M.E., Glen D.M., Kendall D.A., & Milsom N.F. (1976). Predation of overwintering larvae of codling moth (Cydia pomonella (L.)) by birds. Journal of applied ecology, 341-352. |
| 33 | Kristín A. (1994). Food variability of nuthatch nestlings (Sitta europaea) in mixed beech forests: Where are limits of its polyphagy? Biologia, 49 (5): 773-779. |
| 34 | Martínez-Cabello A., Soler M., & Soler J.J. (1991). Alimentación de la Tarabilla común (Saxicola torquata) en el sureste de la Península Ibérica durante el periodo otoño-invierno. Ardeola, 38 (2): 317-326. |
| 35 | Davies N.B. (1977). Prey selection and the search strategy of the spotted flycatcher (Muscicapa striata): a field study on optimal foraging. Animal Behaviour, 25: 1016-1033. |
| 36 | López-Iborra G., Limiñana R., Peñarrubia S.G., Pinheiro R.T. (2005). Diet of common chiffchaffs (Phylloscopus collybita) wintering in a wetland in south-east Spain. Revista Catalana d'Ornotologia, 21: 29-36. |
| 37 | Bouaziz P.M.A. (2013). Quelques aspects sur la bioécologie d’une race orientale du pouillot véloce Phylloscopuscollybita (tristis? ou abientinus?)(Aves, Sylviidae) dans le parc national d’El-Kala. Doctoral dissertation |
| 38 | Pérez-Granados C., Serrano-Davies E. (2016). Reyezuelo listado – Regulus ignicapilla. In: Enciclopedia Virtual de los Vertebrados Españoles. Salvador, A., Morales, M. B. (Eds.). Museo Nacional de Ciencias Naturales, Madrid. http://www.vertebradosibericos.org/ |
| 39 | Bibby C.J. (1979). Bibby, C. J. (1979). Foods of the Dartford warbler Sylvia undata on southern English heathland (Aves: Sylviidae). J. Lond. Zool., 188: 557-576. |
| 40 | Gruar D., Peach W., & Taylor R. (2003). Summer diet and body condition of Song Thrushes Turdus philomelos in stable and declining farmland populations. Ibis, 145: 637-649. |
| 41 | Iglesias D.J., Gil-Delgado J.A., & Barba E. (1993). Diet of Blackbird nestlings in orange groves: seasonal and age-related variation. Ardeola, 40 (2): 113-119. |
| 42 | Aparicio R.J. (2016). Mirlo común – Turdus merula. In: Enciclopedia Virtual de los Vertebrados Españoles. Salvador, A., Morales, M. (Eds.). Museo Nacional de Ciencias Naturales, Madrid. http://www.vertebradosibericos.org/ |
| 43 | Poulsen J.G., Sotherton N.W., & Aebischer N.J. (1998). Poulsen, J. G., Sotherton, N. W., & Aebischer, N. J. (1998). Comparative nesting and feeding ecology of skylarks Alauda arvensis on arable farmland in southern England with special reference to set‐aside. Journal of Applied Ecology, 35: 131-147. |
| 44 | Jenny M. (1990). Diet-ecology of the skylark (Alauda arvensis) in an intensively cultivated agroecosystem in the Swiss Midlands. Der Ornithologische Beobachter, 87: 31-53. |
| 45 | Herranz J., Yanes M., & Suárez F. (1997). La dieta de los pollos de dos aláudidos simpátricos: Cogujada Montesina (Galerida theklae) y Terrera Marismeña (Calandrella rufescens). In: Actas de las XII Jornadas Ornitológicas Españolas: Almerimar (El Ejido-Almería), 15 a 19 de septiembre, 1994 (pp. 123-133). Instituto de Estudios Almerienses. |
| 46 | Motis A., Estrada J., & Oro D. (1997). Nestling diet of the spotless starling Sturnus unicolor and the European starling Sturnus vulgaris in a sympatric breeding area. Ornis Fennica, 74: 179-185. |
| 47 | Peris S. (1980). Biologia del estornino negro (Sturnus unicolor) II. Dieta del pollo. Doñana Acta Vertebrata, 7 (2): 249-260. |
| 48 | Peris S. (1980). Biología del estornino negro (Sturnus unicolor Temm.): 1. Alimentación y variación de la dieta. Ardeola, 25: 207-240 |
| 49 | Benabbas-Sahki I., Bakiri A., Julliard R., & Doumandji E.S. (2013). Wryneck Diet Analysis Jynx torquilla mauretanica Near Algiers (Algeria) Further on Previous Knowledge. International Journal of Bio-Technology and Research, 3 (2): 27-34. |
| 50 | Jiao Z., Wan T., Wen J., Hu J., Luo Y., Fu L., & Zhang L. (2008). Seasonal diet of the Great Spotted Woodpecker (Picoides major) in shelterwood plantations of Wulate Qianqi county, Inner Mongolia. Forestry Studies in China, 10 (2): 119-124. |
| 51 | Moreby S.J., & Stoate C. (2000). A quantitative comparison of neck-collar and faecal analysis to determine passerine nestling diet. Bird Study, 47(3): 320-331. |
| 52 | Tryjanowski P., Karg M.K., & Karg J. (2003). Diet composition and prey choice by the red-backed shrike Lanius collurio in western Poland. Belg. J. Zool., 133 (2): 157-162. |
| 53 | Hódar J.A. (2006). Diet composition and prey choice of the southern grey shrike Lanius meridionalis L. in south-eastern Spain: the importance of vertebrates in the diet. Ardeola, 53(2): 237-249. |
| 54 | Karlsson S. (2004). Season-dependent diet composition and habitat use of red-backed shrikes Lanius collurio in SW Finland. Ornis Fennica, 81: 97-108. |
| 55 | Lepley M., Thevenot M., Guillaume C.P., Ponel P., & Bayle P. (2004). Diet of the nominate Southern Grey Shrike Lanius meridionalis meridionalis in the north of its range (Mediterranean France). Bird Study, 51: 156-162. |
| 56 | Nikolov B.P. (2002). Diet of the red-backed shrike Lanius collurio in Bulgaria. Acrocephalus, 23: 21-26. |
| 57 | Lefranc N., & Issa N. (2013). Plan national d'actions: Pies-grièches, Lanius sp. 2014-2018. Ministère de l’écologie, LPO. |
| 58 | Giannangeli L., de Sanctis A., Maginelli R., & Medina F.M. (2004). Seasonal variation of the diet of the Stone Curlew Burhinus oedicnemus distinctus at the island of La Palma, Canary Islands. Ardea, 92 (2): 175-184 |
| 59 | Amat J.A. (1986). Information on the diet of the stone curlew Burhinus oedicnemus in Donana, southern Spain. Bird Study, 33: 71-73. |
| 60 | Green R.E., Tyler G.A., & Bowden C.G.R. (2000). Habitat selection, ranging behaviour and diet of the stone curlew (Burhinus oedicnemus) in southern England. J. Zool. Lond., 250: 161-183 |
| 61 | Johansson O.C., & Blomquist D. (1996). Habitat selection and diet of lapwing Vanellus vanellus chicks on coastal farmland in SW Sweden. Journal of Applied Ecology, 33: 1030-1040 |
| 62 | Galbraith H. (1987). The diet of lapwing Vanellus vanellus chicks on Scottish farmland. Ibis, 131: 80-84. |
| 63 | Calvo B. (1994). Effects of agricultural land-use on the breeding of Collared Pratincole Glareola pratincola in south-west Spain. Biological Conservation, 70: 77-83. |
| 64 | Inglisa M., Galeotti P, & Taglianti A.V. (1993). The diet of a coastal population of European bee‐eaters (Merops apiaster) compared to prey availability (Tuscany, central Italy). Italian Journal of Zoology, 60(3): 307-310. |
| 65 | Kristin A. (1994). Breeding biology and diet of the bee-eater (Merops apiaster) in Slovakia. Biologia, Bratislava, 49 (2): 273-279. |
| 66 | Fournier J., & Arlettaz R. (2001). Food provision to nestlings in the Hoopoe Upupa epops: implications for the conservation of a small endangered population in the Swiss Alps. Ibis, 143: 2-10. |
| 67 | Gania I.M., Litvak M.D. & Kukurusianu L.S. (1969). Food of some birds from Moldavia. Vopr. fkol. Prakt. Znatchenija Ptits i Mlekopitaivshikh Moldavii 4: 26-54. |
| 68 | Boukhemza M., Doumandji S., Voisin C., & Voisin J.F. (2000). Disponibilités des ressources alimentaires et leur utilisation par le Héron garde-bœufs Bubulcus ibis en Kabylie, Algérie. Revue d'Écologie (Terre Vie), 55: 361-381. |
| 69 | Si Bachir A., Hafner H., Tourenq J.N., Doumandji S., & Lek, S. (2001). Diet of adult cattle egrets (Bubulcus ibis) in a new North African colony (Soummam, Kabylie, Algeria): taxonomic composition and seasonal variability. Ardeola, 48(2): 217-223. |
| 70 | Romanowski J., Altenburg D., & Zmihorski M. (2013). Seasonal variation in the diet of the little owl, Athene noctua in agricultural landscape of Central Poland. North-Western Journal of Zoology, 9 (2): 310-318. |
| 71 | Bon M., Ratti E., & Sartor A. (2001). Variazione stagionale della dieta della civetta Athene noctua (Scopoli, 1769) in una località agricola della gronda lagunare veneziana. Boll. Mus. Civ. St. Nat. Venezia, 52: 193-212. |
| 72 | Ortego J. (2016). Cernícalo primilla – Falco naumanni. En: Enciclopedia Virtual de los Vertebrados Españoles. Salvador, A., Morales, M. B. (Eds.). Museo Nacional de Ciencias Naturales, Madrid. http://www.vertebradosibericos.org/ |
| 73 | Carrillo J., Hernández E.C., Nogales M., Delgado G., García R., & Ramos T. (1994). Geographic variation in the spring diet of Falco tinnunculus L. on the islands of Fuerteventura and El Hierro (Canary Islands). Bonn. Zool. Beitr., 45: 39-48 |
| 74 | Collinger W.E. (1913). The food of some British wild birds: a Study in Economic Ornithology. Dulau & CO., Limited. London. |
| 75 | Cramp S., Simmons K.E.L. (1986). The birds of Western Paleartic. Vol. I: Ostrich to Ducks. |
| 76 | Cramp S., Simmons K.E.L. (1993-1994). The birds of Western Paleartic. Vol. II: Hawks to Bustards. |
| 77 | Cramp S., Simmons K.E.L. (1983). The birds of Western Paleartic. Vol. III: Waders to Gulls. |
| 78 | Cramp S., Simmons K.E.L. (1994). The birds of Western Paleartic. Vol. IV: Terns to Woodpeckers |
| 79 | Cramp S. (1988-90). The birds of Western Paleartic. Vol. V: Tyrant Flycatchers to Thrushes. |
| 80 | Cramp S. (1992). The birds of Western Paleartic. Vol. VI: Warblers |
| 81 | Cramp S. & Perrins C.M. (1993). The birds of Western Paleartic. Vol. VII: Flycatchers to Shrikes. |
| 82 | Cramp S. & Perrins C.M. (1994). The birds of Western Paleartic. Vol. VIII: Crows to Finches. |
| 83 | Cramp S. & Perrins C.M. (1994). The birds of Western Paleartic. Vol. IX: Buntings and New World Warblers. |
| 84 | Vietinghoff-Riesch A.F. von (1924). Das Verhalten paläarktischer Vögel gegenüber den wichtigeren forstschädlichen Insekten, V-IX. Zeitschrift für angewewandte Entomologie, XII, 3: 483-512. |
| 85 | Ceia R.S., & Ramos J.A. (2016). Birds as predators of cork and holm oak pests. Agroforestry systems, 90(1): 159-176. |
| 86 | Kristin A. & Patocka J. (1997). Birds as predators of Lepidoptera: Selected examples. Biologia, Bratislava, 55 (2): 319-326. |
| 87 | Sierro A., & Arlettaz (1997). Barbastelle bats (Barbastella spp.) specialize in the predation of moths: implications for foraging tactics and conservation. Acta Oecologica, 18: 91-106 |
| 88 | Krüger F., Clare E.L., Greif S., Siemers B.M., Symondson W.O.C., & Sommer R.S. (2014). An integrative approach to detect subtle trophic niche differentiation in the sympatric trawling bat species Myotis dasycneme and Myotis daubentonii. Molecular Ecology, 23: 3657-3671 |
| 89 | Vesterinen E.J., Lilley T., Laine V.N., & Wahlberg N. (2013). Next generation sequencing of fecal DNA reveals the dietary diversity of the widespread insectivorous predator Daubenton’s bat (Myotis daubentonii) in Southwestern Finland. PloS one, 8(11): e82168. |
| 90 | Vesterinen E.J., Ruokolainen L., Wahlberg N., Peña C., Roslin T., Laine V.N., ... & Lilley T.M. (2016). What you need is what you eat? Prey selection by the bat Myotis daubentonii. Molecular ecology, 25(7): 1581-1594. |
| 91 | Hope P.R., Bohmann K., Gilbert M.T.P., Zepeda-Mendoza M.L., Razgour O., & Jones, G. (2014). Second generation sequencing and morphological faecal analysis reveal unexpected foraging behaviour by Myotis nattereri (Chiroptera, Vespertilionidae) in winter. Frontiers in Zoology, 11:39 |
| 92 | Racey P.A., & Swift S.M. (1985). Feeding ecology of Pipistrellus pipistrellus (Chiroptera: Vespertilionidae) during pregnancy and lactation. I. Foraging behaviour. The Journal of Animal Ecology: 205-215. |
| 93 | Puig-Montserrat X., Torre I., López-Baucells A., Guerrieri E., Monti M.M., Ràfols-García R., ... & Flaquer C. (2015). Pest control service provided by bats in Mediterranean rice paddies: linking agroecosystems structure to ecological functions. Mammalian Biology, 80: 237-245 |
| 94 | Rydell J., Bogdanowicz W., Boonman A., Petterson S., Suchecka E., Pomorski J. (2016). Bats may eat diurnal flies that rest on wind turbines. Mammalian Biology, 81: 331-339 |
| 95 | Goiti U., Vecin P., Garin I., Saloña M., & Aihartza J.R. (2003). Diet and prey selection in Kuhl’s pipistrelle Pipistrellus kuhlii (Chiroptera: Vespertilionidae) in south-western Europe Acta Theriologica, 48(4): 457-468 |
| 96 | Boreau de Roincé C., Ricard J. M., Garcin A., Jay M., Mandrin J.F., Lavigne C., Bouvier J.C. (2010). Lutte biologique par conservation de la biodiversité: Fonctionnalité des auxiliaires vertébrés et invertébrés dans le contrôle des ravageurs du pommier (1ère partie). Infos CTIFL, 263, 10-15. |
| 97 | Ricard J.M., Jay M., Garcin A. & Mandrin J.F. (2008): Mesure de la prédation des ravageurs par des auxiliaires vertébrés et invertébrés: Développement d’un outil biomoléculaire. Infos-Ctifl, 241: 15–19 (5). |
| 98 | Uhrin M., Kanuch P., Benda P., Hapl E., Verbeek H.D., Kristin A., … & Andreas M. (2006). On the Greater noctule (Nyctalus lasiopterus) in central Slovakia. Vespertilio, 9-10: 183-192. |
| 99 | Shiel C.B., Duvergé P.L., Smiddy P., & Fairley J.S. (1998). Analysis of the diet of Leisler's bat (Nyctalus leisleri) in Ireland with some comparative analyses from England and Germany. J. Zool. London, 246: 417-425 |
| 100 | Jones G. (1990). Prey selection by the greater horseshoe bat (Rhinolophus ferrumequinum): optimal foraging by echolocation? Journal of Animal Ecology, 59: 587-602 |
| 101 | Flanders J., & Jones G. (2009). Roost use, ranging behavior, and diet of greater horseshoe bats (Rhinolophus ferrumequinum) using a transitional roost. Journal of Mammalogy, 90(4): 888-896 |
| 102 | McAney C.M., & Fairley J.S. (1989). Analysis of the diet of the lesser horseshoe bat Rhinolophus hipposideros in the West of Ireland. J. Zool. London, 217: 491-498. |
| 103 | Mitschunas N., & Wagner M. (2015). Diet of the lesser horseshoe bat (Rhinolophus hipposideros) in Central Germany and its seasonal and site-specific variation. Acta Chiropterologica, 17 (2): 379-392. |
| 104 | Mata V.A., Amorim F., Corley M.F., McCracken G.F., Rebelo H., & Beja P. (2016). Female dietary bias towards large migratory moths in the European free-tailed bat (Tadarida teniotis). Biology Letters, 12(3), 20150988. |
| 105 | Whitaker J.O., Shalmon B., & Kunz T.H. (1994). Food and feeding habits of insectivorous bats from Israel. Zeitschrift fur Saugetierkunde, 59(2): 74-81. |
| 106 | Zeale M.R., Butlin R.K., Barker G.L., Lees D.C., & Jones G. (2011). Taxon‐specific PCR for DNA barcoding arthropod prey in bat faeces Molecular Ecology Resources, 11(2): 236-244. |
| 107 | Whitaker J., & Karataş A. (2009). Food and feeding habits of some bats from Turkey. Acta Chiropterologica, 11 (1): 393-403. |
| 108 | Dietz C., Nill D., & von Helversen O. (2009). Bats of Britain, Europe and Northwest Africa. A & C Black. London. |
| 109 | Vaughan (1997). The diets of British bats (Chiroptera). Mammal Review, 27(2): 77-94. |
| 110 | Poulton E.B. (1929). British insectivorous bats and their prey. In: Proceedings of the Zoological Society of London (Vol. 99, No. 2, pp. 277-303). Oxford, UK: Blackwell Publishing Ltd. |
| 111 | Wickramasinghe L.P., Harris S., Jones G., & Vaughan Jennings N. (2004). Abundance and species richness of nocturnal insects on organic and conventional farms: effects of agricultural intensification on bat foraging. Conservation Biology, 18(5): 1283-1292 |
| 112 | Riccucci M., & Lanza B. (2014). Bats and insect pest control: a review. Vespertilio, 17: 161-169. |
